# Pre- and peri-operative clinical information, physiological observations and outcome measures following flexible ureterorenoscopy (FURS), for the treatment of kidney stones. A single-centre observational clinical pilot-study in 51 patients

**DOI:** 10.1186/s12894-022-01053-0

**Published:** 2022-07-14

**Authors:** Stephen Fôn Hughes, Alyson Jayne Moyes, Kevin Jones, Christopher Bell, Abigail Duckett, Ahmed Moussa, Iqbal Shergill

**Affiliations:** 1 North Wales & North West Urological Research Centre, Betsi Cadwaladr University Health Board (BCUHB) Wrexham Maelor Hospital, Wrexham, Wales, UK; 2 Maelor Academic Unit of Medical & Surgical Sciences (MAUMSS), Betsi Cadwaladr University Health Board (BCUHB), Wrexham Maelor Hospital, Wrexham, Wales, UK; 3 School of Medical Sciences, Bangor University, Bangor, Wales, UK; 4 Department of Biological Sciences, University of Chester, Chester, UK; 5 Impact Medical, Aintree Racecourse Retail & Business Park, Liverpool, UK; 6 The Alan de Bolla Department of Urology, BCUHB Wrexham Maelor Hospital, Wrexham, Wales, UK

**Keywords:** Flexible Ureterenoscopy, (FURS), Kidney stones, Clinical outcome measures, Infection, Post-operative complications

## Abstract

**Background:**

Kidney stone disease contributes to a significant proportion of routine urological practice and remains a common cause of worldwide morbidity. The main aim of this clinical-pilot study was to investigate the effect of flexible ureterorenoscopy (FURS) on pre- and peri-operative clinical information, physiological observations and outcome measures.

**Methods:**

Included were 51 patients (31 males, 20 females), who underwent elective FURS, for the treatment of kidney stones.

Pre-operative and peri-operative clinical information, and post-operative physiological observations and outcome measures were collected using a standard case report form. Pre-operative clinical information included age, gender, BMI, previous history of stone formation and hypertension. Pre-operative stone information included the size (mm), Hounsfield units (HU), laterality and intra-renal anatomical location. Peri-operative surgical details included surgical time in minutes; Laser use; Duration and energy of laser; and post-operative stenting. The physiological outcomes measured included systolic and diastolic blood pressure (mmHg), Likert pain score, temperature, heart rate (bpm) and respiration rate (bpm).

Following initial descriptive analysis, a series of Pearson’s correlation coefficient tests were performed to investigate the relationship between surgical factors other variable factors.

**Results:**

A series of significant, positive correlations were observed between; age and surgical time (*p* = 0.014, r = 0.373); stone size and Hounsfield unit (*p* = 0.029, r = 0.406); surgical time and duration of laser (*p* < 0.001, r = 0.702); surgical time and BMI (*p* = 0.035, r = 0.322); baseline heart rate and Hounsfield unit (*p* = 0.026, r = − 0.414); base line heart rate and BMI (*p* = 0.030, r = 0.307).; heart rate at 120-min post FURS and age (*p* = 0.038, r = − 0.308); baseline pain score and BMI (*p* = 0.010, r = 0.361); baseline respiration rate and BMI (*p* = 0.037, r = 0.296); respiration rate at 240-min post FURS and BMI (*p* = 0.038, r = 0.329); respiration rate at 120 min post FURS and age (*p* = 0.022, r = − 0.330). Four patients developed post-operative complications (3—UTIs with urinary retention, 1–urosepsis).

**Conclusions:**

We report that following FURS there is an association between various physiological, clinical and surgical parameters. Although these correlations are weak, they warrant further investigation as these may be linked with untoward complications, such as infection that can occur following FURS. This data, however, will need to be validated and reproduced in larger multi-centre studies.

## Background

In today’s practice, kidney stone disease—medically termed ‘nephrolithiasis’, contributes to a significant proportion of routine urological practice and remains a common cause of worldwide morbidity [1, 2].

The effects of nephrolithiasis are global, with the average lifetime risk of developing the condition being 5–10% [3]. However, industrialized countries exhibit higher incidence rates, with the male lifetime risk of developing renal calculi being 18.8% and the female risk being 9.4% [4]. Alarmingly, both the incidence and prevalence of kidney stones (renal calculi) continues to rise, irrespective of age, race or gender [5–8]. This high incidence rate (> 10%) masks the burden of continuing morbidity, as 50% of patients with nephrolithiasis experience stone recurrence within 5–7 years [3]. More specifically it is estimated that approximately 10% of people who have experienced an episode of renal calculi will have an episode of reoccurrence within one year, 35% within five years and 50% within ten years [9]. This growth in incidence brings a multitude of associated issues, both financially to healthcare providers and socially and economically to patients [10].

Flexible ureterorenoscopy (FURS) is considered a standard surgical treatment option for kidney stones. The overall complication rate after FURS is 9–25%, with problems such as infection, bleeding, renal injury, sepsis, haematuria and pain being the most reported [11, 12]. In addition to this, the passage of scopes has also been documented to cause ureteric and renal trauma, in some instances leading to mucosal ischaemia following FURS [13]. Consequently, it can be appreciated that disturbances to the normal vascular integrity due to the passage of scopes and the application of neodymium-doped yttrium aluminium garnet (YAG) holmium laser could explain alterations to haematological, biochemical, inflammatory and endothelial profiles, and the resultant instances of haemorrhages or thromboembolic complications observed at the site of injury in some patients [14, 15].

Assessing clinical outcome by monitoring patients’ vital signs is a well-established practice in healthcare. More specifically the observation of vital signs provides an integral and important insight into a patient’s wellbeing and any deterioration of status [16]. Early warning systems have seen widespread use across the UK for a number of years, with pulse, respiratory rate, systolic blood pressure, temperature and pain score being commonly recorded [17]. Abnormalities in observed vital signs can indicate a disease state or abnormal physiological changes within a patient with different vital signs highlighting different problem areas [16]. For example, if a patient presents with pyrexia (body temp > 38 °C) after 48 h post-operatively, infectious causes are considered as the most likely culprit. However, if patients exhibit pyrexia within the first 48 h post-surgery it can, in most cases, be recognised as being non-infectious in origin [18]. A fever occurring immediately post-operatively is common, and usually occurs as a result of increased circulating pyrogenic cytokines including interleukins (IL) IL-1, IL-6, tumour necrosis factor and interferon-γ. The release of these cytokines is core to the inflammatory response, tissue repair and normal healing [18].

Variation in vital signs 24 h prior to discharge have been shown to be associated with an increased risk of adverse post-discharge outcomes for patients [19]. Specifically, it has been found that patients discharged with unstable vital signs can have as much as a 40% higher risk of death or readmission within 30 days of discharge than patients discharged with stable vital signs [19]. Therefore, it is imperative to monitor vital signs in postoperative patients to detect any deterioration to physiological function, although it is prudent to remember that physiological parameters can be disturbed due to the acute phase postoperative inflammatory responses, and may not result in an progressive clinical decline [20].

With regards to pharmacological consideration following FURS, best clinical practice guidelines set out by the European Association of Urology (EAU) state that patients undergoing FURS are sedated using predominately general anaesthesia, although the use of spinal or local anaesthesia is sometimes appropriate [21]. Shaikh, Khalid & Zaidi (2008) compared FURS carried out under general anaesthesia (*n* = 30) verse spinal anaesthesia (*n* = 30), [22]. Their results suggest that FURS procedures carried out under spinal anaesthesia correlated to a reduced surgical time, with general anaesthesia averaging 30.5 ± 2.13 min vs FURS under spinal anaesthesia which took an average of 14.4 ± 1.29. Furthermore, patients under spinal anaesthesia had an overall decrease in the duration of their hospital stay vs those under general anaesthesia (21.6 h vs 18.1 h respectively) [22], yet general anaesthesia is still predominantly used in today’s urological practice. Pain management via the use of non-steroidal anti-inflammatory drugs (NSAIDs) are a staple for treating symptoms of acute renal colic in patients in the run up to their surgical intervention. However, these medications are well documented for their role in reducing inflammation and may therefore interfere in the post-operative natural healing process [23, 24].

We have previously reported on the safety and efficacy and changes to routine and novel biomarkers following various urological and other surgical procedures [25–32]. The present study intends to build on previous work and to consider the association between various physiological, surgical and clinical outcome measures following FURS.

Specifically, the main aim of this clinical-pilot study was to investigate the effect of FURS on pre- and peri-operative clinical information, physiological observations and outcome measures. It is envisaged that the results of this study will contribute new knowledge to the field, which ultimately may aid clinicians in the management of their patients.

## Methods

### Subject volunteers

Ethical approval for this study was received from the Welsh Research Ethics Service (REC) 4 committee (REC4: 12/WA/0117) and were carried out in accordance with the ethical rules of the Helsinki Declaration and Good Clinical Practice. Fifty-one consecutive patients undergoing elective FURS for the treatment of kidney stones were recruited (*n* = 51) after written informed consent. Of these, 31 were males and 20 were females, aged between 28—87 years (median 50 years).

### Flexible ureterorenoscopy (FURS)

FURS was performed as per local protocol at the Betsi Cadwaladr University Health Board (BCUHB) Wrexham Maelor, NHS Hospital, North Wales, UK under the same consultant urologist. Namely, under General Anaesthesia, using Olympus P6 Flexible ureterorenoscope, stone fragmentation was performed using Auriga XL laser at initial settings of 5 Hz and 500 mJ (2.5 W energy) and increasing until adequate stone fragmentation for retrieval using Boston Scientific 1.9Fr Zero tip basket.

### Clinical information and physiological outcomes

Pre-operative and peri-operative clinical information, and post-operative physiological observations and outcome measures were collected using a standard case report form (CRF). Pre-operative clinical information included age, gender, BMI, previous history of stone formation and hypertension. Pre-operative stone information included the size (mm), Hounsfield units (HU), laterality (left, right, bilateral) and intra-renal anatomical location (upper-pole, mid-pole or lower-pole). Peri-operative surgical details included (1) surgical time in minutes; (2) Laser use, stating simply whether laser was used during the procedure to help fragment stones; (3) Duration (time in minutes) and energy of laser (joules) used; and (4) Post-operative stenting, indicating whether or not patients had a temporary stent fitted prior to or during the procedure.

Where stone fragments could be obtained, intra-operatively, evaluation of stone composition was carried out by infrared spectroscopy analysis by trained specialist biochemists at the Leicester Royal Infirmary (UK).

The physiological outcomes measured include, systolic and diastolic blood pressure (mmHg), Likert pain score ranging from 1 (lowest pain score) to 5 (highest pain score), temperature using temporal artery device (^O^C), heart rate (bpm) and respiration rate (bpm). These were documented prior to surgery (baseline) and at 30, 120, 240 min post operatively.

### Statistical analysis

Statistical analysis was undertaken using the latest version of SPSS (26.0). Initially, descriptive statistics (e.g. mean, median, range, etc.) and testing for normality was carried out*.* Where data normally distributed, parametric analysis, employing repeated measures one-way analysis of variance (ANOVA) between samples test was used, adopting a 5% level of significance. Post hoc testing was conducted using the Bonferroni test for pairwise comparisons between means. All parametric data is presented as mean ± S.D. Where data did not comply with normality, the non-parametric equivalent tests were used.

Following initial analysis, a series of Pearson’s correlation coefficient tests were performed as appropriate, to investigate the relationship between surgical factors (i.e. duration of laser, stone size surgical time, Hounsfield unit, BMI and age). Additionally, further correlations were carried out to establish potential interactions amongst other variable factors (e.g. age and stone size). Interactions were found to be significant at the *p* ≤ 0.05 confidence limit (2-tailed). The size of correlation was interpreted using the categories outlines in Table 1.

**Table 1:** Approach for Interpreting the Size of a Correlation Coefficient (Adapted from Mukaka 2012 [33])

Size of Correlation		Interpretation
Positive	Negative	
0.90 to 1.00	− 0.90 to − 1.00	Very high positive (or negative) correlation
0.70 to 0.90	− 0.70 to − 0.90	High positive (or negative) correlation
0.50 to 0.70	− 0.50 to − 0.70	Moderate positive (or negative) correlation
0.30 to 0.50	− 0.30 to − 0.50	Low positive (or negative) correlation
0.00 to 0.30	0.00 to − 0.30	negligible correlation

## Results

Four of the 51 patients in the study were diagnosed with a post-operative complication following FURS. These four patients were re-admitted to hospital within 48 h of discharge following their day case procedures. These patients are identified as being participant 9, 10, 12 and 32. Participants 9, 10 and 32 were male and participant 12 is female. Retrospective observation of their medical notes shows that participants 9, 10 and 12 were re-admitted due to developing an acute urinary tract infection with urinary retention, and participant 32 was treated for urosepsis.

### Patients’ baseline characteristics

Information provided in Table 2, represents patients baseline (pre-operative) characterises to include, age, gender, BMI, stone formation history, blood pressure, stone location, number of stones, and stone largest dimensions.

**Table 2:** Patients baseline (pre-operative) characteristics, (*n* = 51)

Age (years)	
Mean	54
Median	50
Range	27–87
*Gender (n = 51)*	
Male	31
Female	20
*Height (m)*	
Mean	1.69
Median	1.7
Range	1.5–1.86
*BMI (kg/m*^*2)*^* category*	
Underweight (< 18.5)	1
Normal weight (18.5–24.9)	18
Overweight (25.0–29.9)	14
Obese class I (30.0–34.9)	10
Obese class II (35.0–39.9)	5
Obese class III (≥ 40.0)	3
*Stone formation history*	
First time stone former	24
Reoccurring stone former	27
*BP prior to procedure (baseline)*	
Normal (< 120/80)	17
Prehypertension (120–39/80–89)	29
HTN stage 1 (140–159/90–99)	5
iStone location	
Left kidney	22
Right kidney	25
Bilateral	4
Upper pole	16
Mid pole	19
Lower pole	16
*Number of stones present*	
*1*	38
2	9
3	4
*Stone largest dimensions (mm)*	
Mean	6
Median	9.17
Range	2.0–25.0

*FURS* flexible ureteroscopy; *BMI* body mass index; *BP* blood pressure; *HTN* hypertension

### Surgical Details

Information provided in Table 3, represents summary of surgical data collected (e.g. duration of FURS procedure, stone samples collected, density of stones, etc.). Stone clearance was achieved in all patients (100% success rate). Stone free rates were documented at 3 month follow up using CT scan, KUB XRay or USS imaging as appropriate. Stone free rates were 100% in all cases.

**Table 3:** Summary of surgical data, (*n* = 51)

Duration of FURS procedure (minutes)	
Mean	58
Median	49
Range	22–104
*Laser used*	
Yes	42
No	9
*Duration of laser use (minutes)*	
Mean	16.8
Median	10.8
Range	0.3–54.2
*Laser Energy (J)*	
Mean	3232.7
*Laser pulse*	
Mean	6272.8
*JJ stent*	
No	18
Presented with *in-situ* stent	8
Stent inserted day of procedure	25
*Stone sample collected*	
Yes	34
No	17
Mean stone size	11
*Subsequent results from stone analysis*	
*Main constituent of stone(s)*	*Patients*
Calcium oxalate monohydrate	14 (48%)
Calcium oxalate dihydrate	2 (7%)
Calcium phosphate	4 (21%)
Cystine	4 (14%)
Calcium hydroxyl phosphate	3 (10%)
*Post-operative complication*	
Yes	4 (UTI *n* = 3, Urosepsis *n* = 1)
No	47

*FURS* flexible ureteroscopy; *DJ Double-J*

Table 4 shows some of the baseline characteristics and surgical data of those patients experiencing post-operative complications, for comparison with the equivalent data for the whole cohort in Tables 2 and 3.

**Table 4:** Baseline characteristics and surgical data for patients with post-operative complications

Parameter	Participant 9	Participant 10	Participant 12	Participant 32
Ranges (27–87 years)	66	50	51	67
BMI (kg/m^2^)	23.7 (normal weight)	23.2 (normal weight)	42.6 (obese class III)	43.2 (obese class III)
Duration of FURS procedure (minutes)	28	32	98	53
Duration of laser use (minutes)			14.1	22.28
Stone size (mm)	2 mm, 20 mm and 5 mm (3 stones)	20 mm (1 stone)	20 mm and 17 mm (2 stones)	18 mm (1 stone)
Stone composition	100% calcium oxalate monohydrate	80% calcium phosphate, 20% calcium oxalate monohydrate	100% calcium phosphate	80% calcium oxalate monohydrate, 20% calcium oxalate dihydrate

### Clinical outcome measurement of vital signs

Vital signs were recorded for all patients undergoing FURS that were recruited to this study (*n* = 51). The results presented in Table 5 illustrate that minimal changes to heart rate, body temperature and respiration rate following FURS. Interestingly, however, it was noted that during the post-operative time course, the average pain score increased with each time increment, from 1.5 at baseline, 1.8 at 30 min, 2.1 at 120 min and peaked at to 2.2 at 240 min post-operatively. Although there were trends of increasing pain following FURS, these changes were not statistically significant (*p* > 0.05).

**Table 5:** Clinical outcome measures observed in FURS patient cohort

Vital sign	Baseline	30 min post-op	120 min post-op	240 min post-op	*P* value
*Heart Rate (per min)* No complications only	75 ± 11	76 ± 17	74 ± 14	72 ± 12	0.084
Participant 9 (UTI)	72	88	64	86	
Participant 10 (UTI)	73	75	61		
Participant 12 (UTI)	100	80	82		
Participant 32 (urosepsis)	77	85	82	97	
*Body Temperature (*^*o*^*C)* No complications only	36.3 ± 0.5	36.4 ± 0.4	36.4 ± 0.6	36.4 ± 0.7	0.237
Participant 9 (UTI)	36.2	36.2	35.5	36.6	
Participant 10 (UTI)	36.5	36.4	36.7		
Participant 12 (UTI)	37.2	36.1			
Participant 32 (urosepsis)	36.5	37.1	37.0	37.5	
*Respiration rate (per min)* No complications only	15 ± 3	16 ± 3	16 ± 2	16 ± 2	0.095
Participant 9 (UTI)	13	13	16	17	
Participant 10 (UTI)	16	14	19		
Participant 12 (UTI)	17	23	18		
Participant 32 (urosepsis)	16	16	16	16	
*Pain Score (1–5)* No complications only	1.5 ± 0.7	1.8 ± 0.9	2.1 ± 1.0	2.2 ± 0.9	0.165
Participant 9 (UTI)	1	1	1	1	
Participant 10 (UTI)	2	4	4		
Participant 12 (UTI)	2	1	3		
Participant 32 (urosepsis)	1	1	1	4	

Data analysed via ANOVA testing, results presented as mean ± standard deviation (n = 51)

Pain score, 1: low, 2.5 moderate, 5 high/extreme pain

### Correlation between patient demographic and surgical parameters

Table 6 reports the analysis of the relationship between; age, stone size, Hounsfield unit, surgical time, duration of laser and BMI.

**Table 6:** Associations (correlation) between surgical parameters and patient demographic data (*n* = 51)

*Correlation*	R Value	P value (2-tailed)
Age and Stone size	− 0.046	0.791
Age and Hounsfield unit	− 0.014	0.942
Age and Surgical time	0.373 (low positive correlation)	0.014*
Age and Duration of laser	0.205	0.278
Age and BMI	0.208	0.147
Stone size and Hounsfield unit	0.406 (low positive correlation)	0.029*
Stone size and Surgical time	0.084	0.676
Stone size and Duration of laser	0.100	0.683
Stone size and BMI	− 0.129	0.466
Hounsfield unit and Surgical time	0.123	0.567
Hounsfield unit and Duration of laser	− 0.002	0.993
Hounsfield unit and BMI	0.148	0.434
Surgical time and Duration of laser	0.702 (high positive correlation)	< 0.001**
Surgical time and BMI	0.322 (low positive correlation)	0.035*
Duration of laser and BMI	0.180	0.342

Person product-moment correlation coefficient

*NB* Please refer to Tables 3 and 4 for the representative data used for the above analysis

*Correlation is significant at the 0.05 level (2-tailed)

** Correlation is significant at the 0.01 level (2-tailed)

Following correlative analysis, subgroup analysis based on; gender (male vs female), age (< 65 vs > 65) and BMI (< 24.9 vs > 24.9) was carried out. No significance was found (*p* > 0.05) for any interactions, indicating that the effect of the intervention on the outcome does not differ within subgroups.

The results presented in Table 6 illustrate that there is a low positive correlation between age and surgical time, implying that as age increases so does the surgical duration (*p* = 0.014, r = 0.373). Interestingly a positive, low correlation between surgical time and BMI was also noted (*p* = 0.035, r = 0.322), indicating that as the BMI increases so does the surgical duration. These findings suggest that both elderly patients and those with increasing BMI’s may be considered a higher risk surgical group for FURS treatment.

Significant interactions were noted between stone size and Hounsfield unit, a low positive correlation was found to be statistically significant (*p* = 0.029, r = 0.406), suggesting that the larger the kidney stone, the denser it is. Furthermore, a high correlation was seen between surgical time and duration of laser use (*p* < 0.001, r = 0.702), indicating that the longer the use of laser, the longer the surgical procedure will take overall.

No significant association (*p* > 0.05) was observed between age and stone size, age and Hounsfield unit, age and duration of laser, age and BMI, stone size and surgical time, stone size and duration of laser, stone size and BMI, Hounsfield unit and surgical time, Hounsfield unit and duration of laser, Hounsfield unit and BMI, or duration of laser and BMI.

### The effect of surgical parameters, age and BMI on vital signs

The results presented in Table 7 show that there is a moderate negative correlation between baseline heart rate and Hounsfield unit (*p* = 0.026, r = -0.414). A low, positive correlation was noted between basal heartrate and BMI (*p* = 0.030, r = 0.307). Furthermore, the heart rate at 120-min post FURS demonstrated a low, negative correlation with age (*p* = 0.038, r = -0.308) suggesting that heart rate declined with increasing age.

**Table 7:** Associations (correlation) between surgical parameters and vital signs at four observation time-points (*n* = 51)

	Duration of Laser		Stone size		Surgical time		Hounsfield unit		BMI		Age (years)	
	r value	*p* value	r value	*p* value	r value	*p* value	r value	*p* value	r value	*p* value	r value	*p* value
*Heart rate (per minute)*												
Baseline	0.144	0.448	− 0.146	0.410	− 0.052	0.740	− 0.414	0.026*	0.307	0.030*	0.013	0.931
30 min post op	− 0.046	0.811	− 0.140	0.446	− 0.060	0.705	− 0.040	0.835	0.134	0.362	− 0.195	0.184
120 min post op	0.092	0.648	− 0.326	0.078	0.093	0.568	− 0.175	0.381	0.107	0.479	− 0.308	0.038*
240 min post op	0.226	0.311	− 0.229	0.240	− 0.006	0.972	− 0.114	0.580	0.057	0.726	− 0.077	0.636
*Pain score*												
Baseline	0.162	0.391	0.043	0.807	0.038	0.808	0.080	0.675	0.361	0.010*	− 0.027	0.855
30 min post op	0.021	0.914	0.094	0.609	− 0.108	0.496	− 0.129	0.504	0.048	0.744	− 0.035	0.812
120 min post op	− 0.103	0.610	− 0.142	0.456	− 0.138	0.396	− 0.195	0.329	− 0.171	0.257	− 0.065	0.668
240 min post op	0.241	0.280	− 0.370	0.053	0.057	0.749	− 0.334	0.096	0.141	0.387	− 0.024	0.883
*Respiration rate (per minute)*												
Baseline	0.066	0.728	0.081	0.649	0.108	0.489	0.149	0.433	0.296	0.037*	− 0.002	0.989
30 min post op	− 0.078	0.686	− 0.149	0.417	− 0.039	0.806	− 0.098	0.615	0.274	0.059	− 0.330	0.022*
120 min post op	− 0.235	0.238	− 0.001	0.996	− 0.141	0.387	− 0.206	0.302	0.06	0.691	− 0.185	0.219
240 min post op	0.228	0.307	0.017	0.931	− 0.092	0.605	− 0.340	0.089	0.329	0.038*	− 0.141	0.386
*Body temperature (*^*0*^*C)*												
Baseline	− 0.150	0.430	− 0.073	0.685	− 0.077	0.629	− 0.009	0.961	− 0.215	0.138	0.212	0.139
30 min post op	− 0.212	0.269	− 0.111	0.544	− 0.213	0.176	0.003	0.990	− 0.240	0.100	0.001	0.997
120 min post op	− 0.280	0.158	− 0.073	0.701	− 0.178	0.272	− 0.098	0.625	− 0.150	0.319	− 0.103	0.495
240 min post op	− 0.174	0.439	− 0.108	0.584	− 0.069	0.698	− 0.207	0.309	− 0.079	0.630	− 0.164	0.312

Person product-moment correlation coefficient

*NB* Please refer to Tables 3–5 for the representative data used for the above analysis

*Correlation is significant at the 0.05 level (2-tailed)

**Correlation is significant at the 0.01 level (2-tailed)

A significant, low, positive correlation was observed between baseline pain score and BMI (*p* = 0.010, r = 0.361) indicating that the higher the BMI the more pain the patients perceived. With regards to respiration rate, there was a positive, negligible association observed between baseline respiration rate and BMI (*p* = 0.037, r = 0. 296). However, a further correlation was observed between the 240-min post FURS respiration rate and BMI, with a low positive correlation (*p* = 0.038, r = 0.329).

Finally, there was a negative, low correlation observed between respiration rate at 120 min post op and age (*p* = 0.022, r = -0.330), suggesting that respiration rate declined as age increased. No correlations were observed between body temperature and surgical parameters.

### Pharmacological considerations

Patients underwent general anaesthesia using either Propofol or Alfentanil intravenous (IV) infusion. 4 patients (15, 16, 21 and 41) were identified as being at an increased risk of post-operative infections and were subsequently prescribed pre-operative antibiotic prophylaxis with 120 mg of intravenous gentamycin. Additionally, patient 32 (who subsequently developed post-operative urosepsis) was prescribed 120 mg of IV gentamycin at 120 min post FURS, and patient 19 was prescribed 500 mg of oral amoxicillin at 240 min post operatively.

Pain was a key and common post-operative symptom noted amongst the whole study population (*n* = 51). In order to minimise patients’ pain, all patients received 1 g of intravenous paracetamol during the procedure. Further pharmacological management of post-operative pain was required for some patients, and this was achieved using varying medications, depending on each individual patients’ needs and risk factors. The medications prescribed for pain management included Morphine, Oramorph, Pethidine, Tramadol, Ketorolac and Codeine in varying doses.

Some patients experienced some additional side-affects following the FURS procedure and were prescribed medications to overcome symptoms. This included nausea, which was treated with Cyclizine (50 mg IV) or Ondansetron (8 mg IV). Relief for abdominal cramping was managed with the administration of 20 mg of Hyoscine butylbromide. Finally, some patients also required management of hypertension and this was achieved using either Ramipril (2.5 mg), Lisinopril (10–80 mg) or Spironolactone (50-100 mg). Table 8 summarises the main findings of this study.

**Table 8:** Summary of main findings, (*n* = 51)

*A series of significant, positive correlations were observed, as summarised below:*
Age and surgical time (*p* = 0.014, r = 0.373)
Stone size and Hounsfield unit (*p* = 0.029, r = 0.406),
Surgical time and duration of laser (*p* < 0.001, r = 0.702)
Surgical time and BMI (*p* = 0.035, r = 0.322)
Base line heart rate and Hounsfield unit (*p* = 0.026, r = -0.414)
Base line heart rate and BMI (*p* = 0.030, r = 0.307)
Heart rate at 120-min post FURS and age (*p* = 0.038, r = -0.308)
Baseline pain score and BMI (*p* = 0.010, r = 0.361)
Baseline respiration rate and BMI (*p* = 0.037, r = 0. 296)
Respiration rate at 240-min post FURS and BMI (*p* = 0.038, r = 0.329)
Respiration rate at 120 min post FURS and age (*p* = 0.022, r = -0.330)
No Significant changes noted with regards to heart rate, respiratory rate, pain score or temperature post FURS for the treatment of kidney stones

## Discussion

The main aim of this clinical-pilot study was to investigate the effect of flexible ureterorenoscopy (FURS) on pre- and peri-operative clinical information, physiological observations and outcome measures.

The results of this study show that there were no significant changes to body temperature, respiration rate, pain score and heart rate from baseline vs 30, 120, 240 min post-FURS. Although not statistically significant, an increasing trend in post-operative pain was recorded, increasing from baseline at 30, 120, and 240 min post op. Literature suggests that pain is a key symptom of renal colic in patients with nephrolithiasis [24]. The finding that pain increases as time progresses, fits with the wider understanding that postoperatively patients will experience pain as a result of trauma to soft tissues, causing an inflammatory response and eliciting an altered neuronal response [34]. Factors that are recognised as impacting the degree and severity of post-operative pain can also include patients’ previous surgical experiences and the mental preparedness of the patient, pain management intra-operatively, nature of surgery and surgical time [35, 36].

The changes observed in regard to pain score may be due to the ongoing effects of Propofol. Propofol is a fast acting IV agent with general anaesthetic and sedative effects, and a relatively short duration of action [37]. Given that the effective half-life of Propofol is 60–120 min, it may explain why patients experience only minimal increases in pain immediately post FURS. This trend of reduced perception of pain following surgery, in patients anaesthetised with Propofol is supported by the findings of other studies. For example, the work by Li et al. (2012) showed that patients undergoing elective laparoscopies that were anaesthetised with Propofol, demonstrated a significantly lowered post-operative pain score at 30 min and 60 min post operatively, when compared to patients anaesthetised with Sevoflurane [38]. The increase in pain displayed in this present study may be due to the diminishing mode of action of Propofol, and as such breakthrough pain could be being experienced with increasing intensity as the agent is metabolised.

Another factor worth considering is the location of the pain felt. In some cases, it could be suggested that the pain felt post-operatively isn’t due to renal colic or surgical trauma but instead due to other external factors, such as positioning during the procedure, or other ongoing medical issues that have been masked by the pain of the calculus. Interestingly, correlative analysis in this present study, found that there was a significant correlation between baseline pain score and BMI (*p* = 0.010, r = 0.361). This may suggest that patients with a higher BMI present with increased pain pre-operatively. Furthermore, studies have shown that sensitivity to pain in obese patients could be due to a pro-inflammatory state. More specifically, tumour necrosis factor-α (TNF-α) and intereukin-6 (IL-6) have shown to be vital chemical mediators in the transmission of pain [39, 40].

Specifically, a significant correlation between stone size (mm) and Hounsfield unit (*p* = 0.029, r = 0.406), was observed in the FURS cohort. A recent study by Kuroda et al. (2018) on 472 FURS patients, demonstrated a statistical correlation between surgical time and stone volume (r = 0.417, *p* < 0.001) and between stone size and Hounsfield unit (r = 0.323, *p* < 0.001) [41]. The results of this study directly relate to our findings, and as such provide insightful clinical data, as both stone size and stone density can impact on surgical length leading to potential complications as reported by others [42, 43]. Furthermore, a recent investigation into FURS and rates of infection, found that the stone volume (mm^3^) is strongly correlated with the risk of developing infections after FURS (*p* = 0.007) [43]. When explored in more detail it was seen that the median stone volume in patients without infections was 357 mm^3^, whereas patients in the group with infections, had a median stone volume of 1,090 mm^3^. This confirms a part of the conclusion drawn by Fan et al. (2015), that stone size is a risk factor for infectious complications after FURS [42]. Similar findings were reported in the present study, in that the 4 patients who developed post-operative complications had larger stones (20 mm, 20 mm, 20 mm, and 18 mm) compared to the rest of the participants who did not develop postoperative problems (mean = 11 mm).

Several significant correlations were noted involving surgical factors including; surgical time and duration of laser, age and surgical time and surgical time and BMI. The positive correlation between surgical time and duration of laser (*p* < 0.001, r = 0.702), is rather self-explanatory. With regards to the positive correlation seen between age and surgical time (*p* = 0.014, r = 0.373), it is appreciated that the duration of the procedure is generally increased in older patients. In principle this correlation sounds unsurprising, likely due to taking a little more care on the older, often more fragile patient. However, a few studies have demonstrated that an increased surgical duration, particularly in older patients is detrimental to health and may result in a higher risk of postoperative delirium, and postoperative cognitive dysfunction (POCD) [44]. Observations from the present study reports that patients who developed complications were into their 6th and 7^th^ decade, although this did not coincide with increased duration of surgical time in our study.

When exploring the positive correlation between surgical time and BMI (*p* = 0.035, r = 0.322), it suggests that the duration of the procedure is generally increased in patients with a higher BMI. A recent study by Raja et al. (2017) explored the effect of BMI in 167 patients undergoing laparoscopic cholecystectomies. The results of their study focus on the average surgical time and reported that the mean surgical time was 75 min for those with a healthy BMI, whereas the surgical time increased to > 90 min in patients with a BMI > 40 [45]. With regards to the FURS procedure, EAU guidelines recommend the use of FURS over SWL in obese patients [21]. Therefore, despite the surgical time being extended, it is unlikely to result in a poorer patient outcome.

Interestingly, there were several positive correlations associated with BMI; between baseline heart rate and BMI (*p* = 0.030, r = 0.307), as well as baseline respiration rate and BMI (*p* = 0.037, r = 0. 296). These results do not represent novel findings, as it is well documented that an increased BMI corresponds with a generally higher heart rate and subsequent respiration rate. However, the impact of this could be clinically relevant, with obesity being shown to cause an impairment in the autonomic nervous system (ANS) [46], in addition to a decrease in parasympathetic modulation and in some case sympathetic modulation [47]. Furthermore, obesity is associated with increased work of breathing because of amplified airways resistance and reduced respiratory system compliance [48]. Additionally, lung volume falls as a result of obesity attributed to the increased abdominal volume and presence of visceral fat [49]. During the FURS procedure, patients are placed in a supine position, however this can exacerbate respiratory problems, leading to a negative impact on the pulmonary mechanics, due to diaphragmatic impedance of the abdomen following the change in lung volume [50]. Therefore, the results of this study may provide new knowledge and guidance to this area and suggests that patients with an increased BMI (> 25 kg/m^2^) should be considered for more intense post-operative monitoring. Interestingly, 2/4 patients who developed post-operative complications in the present study were obese (BMIs > 40.0), and this finding provides an ideal platform that warrants future studies that can investigate the associations between BMI and post-operative complications in a wider ranging context.

The lack of a significant correlation in the other parameters measured (Tables 5 and 6) could be due to the timings at which the observations were recorded. Although a baseline (pre-operative) set of observations were taken these did not show patient parameters in health. in the baseline observations all patients were suffering with the nephrolithiasis. As such observations such as, pulse, respiratory rate and systolic blood pressure may have been altered because of pain or analgesia. Subgroup analysis based on; gender, age and BMI were carried out in this study. No statistical significance was found, concluding that the effect of the intervention on the outcome, does not differ within subgroups. However, it is envisaged that with increased cohort sizes that changes will be present, and as such this should be considered for subsequent and future investigations involving multiple centres.

It is acknowledged that a limiting factor associated with study is the limited budget, the relatively small number of patients recruited, and the lack of subsequent post-operative information available beyond the assessed time points (i.e. up to 240 min). This was difficult to achieve as all the operations were day-case procedures. However, the significant observations and subsequent associations made in the present study between various physiological, clinical and surgical parameter will no doubt provide useful information and may aid the management of FURS patients, in the future.

## Conclusions

We report that following FURS there is an association between various physiological (e.g., BMI), clinical (e.g. stone size) and surgical (e.g. duration of procedure) parameters. Although these correlations are weak, they warrant further investigation as these may be linked with untoward complications such as infection that can occur following FURS. This data, however, will need to be validated and reproduced in larger multi-centre studies.

## Data Availability

The datasets used and/or analysed during the current study are available from the corresponding author on reasonable request.

## Abbreviations

BMI: Body mass index
BP: Blood pressure
FURS: Flexible ureteroscopy
HTN: Hypertension
HU: Hounsfield units
SD: Standard deviation
UTI: Urinary tract infection
